# Clinical Investigation of the Role of Interleukin-4 and Interleukin-13 in the Evolution of Prostate Cancer

**DOI:** 10.3390/cancers3044281

**Published:** 2011-12-16

**Authors:** Robert Goldstein, Charles Hanley, Jonathan Morris, Declan Cahill, Ashish Chandra, Peter Harper, Simon Chowdhury, John Maher, Sophie Burbridge

**Affiliations:** 1 Department of Research Oncology, King's Health Partners Integrated Cancer Center, King's College London, Guy's Hospital Campus, Great Maze Pond, London SE1 9RT, UK; E-Mails: drgoldstein05@yahoo.co.uk (R.G.); 0606723h@student.gla.ac.uk (C.H.); jonathan.morris@kcl.ac.uk (J.M.); 2 Guy's and St. Thomas' NHS Foundation Trust, Great Maze Pond, London SE1 9RT, UK; E-Mails: declan.cahill@gstt.nhs.uk (D.C.); ashish.chandra@gstt.nhs.uk (A.C.); peter@harper.uk.com (P.H.); simon.chowdhury@gstt.nhs.uk (S.C.); 3 Department of Immunology, Barnet and Chase Farm NHS Trust, Barnet, Hertfordshire, EN5 3DJ, UK; E-Mail: john.maher@kcl.ac.uk (J.M.); 4 Department of Clinical Immunology and Allergy, King's College Hospital NHS Foundation Trust, Denmark Hill, London SE5 9RS, UK

**Keywords:** prostate cancer, interleukin-4, interleukin-13

## Abstract

Prostate cancer is the most common cancer in men, both in the USA and Europe. Although incurable, metastatic disease can often be controlled for years with anti-androgen therapy. Once the disease becomes castrate resistant, the median survival is 18 months. There is growing evidence that the immune system, and in particular cytokines, play an important role in prostate cancer immunosurveillance and progression. Here, we have undertaken a clinical investigation of the role of two closely related cytokines, IL-4 and IL-13 in prostate cancer. In the largest series studied to date, we show that serum IL-4, but not IL-13 is significantly elevated in castrate resistant, compared to androgen sensitive disease. Notably however, serum IL-4 levels are also raised in patients with benign prostatic disease. Analysis of benign and malignant prostate tissue demonstrates that the source of IL-4 is epithelial cells rather than infiltrating leukocytes. Together, our data are consistent with a dual role for IL-4 in prostate cancer development. In benign disease, our data add to the evidence that IL-4 serves a protective role. By contrast, the data support a direct role for IL-4 in the progression of prostate cancer from androgen responsive, to advanced castrate-resistant disease.

## 1. Introduction

Prostate cancer is the most common male cancer in the USA and Europe. In its earliest stage, the disease is curable with radical surgery or radiotherapy. While metastatic disease cannot be eradicated, it can often be controlled for years with anti-androgen therapy. In that setting however, evolution to castrate resistance is inevitable and median survival is then only 18 months. Greater understanding of the mechanisms that underlie disease progression are of clear interest. Growing evidence suggests that interleukin (IL)-4 may play an important role in this transition [1,2].

Interleukin-4 is a pleiotropic cytokine produced largely by activated Th2-polarized T-cells, mast cells and basophils. It acts upon a broad range of targets, including hematopoietic cells, endothelial cells and tumor cells. Interleukin-4 interacts with two distinct heterodimeric receptors, both of which share the high affinity IL-4Rα subunit [3]. In the type 1 receptor, the IL-4Rα subunit undergoes IL-4-dependent dimerization with the γ^c^ chain [4]. In addition, IL-4Rα can pair with the IL-13Rα subunit to form the type 2 receptor complex and this process may be driven either by IL-4 or the closely related cytokine, IL-13 [5,6]. Since γ^c^ chain is expressed in hematopoietic cells only, the type 1 heterodimer acts as the receptor for IL-4 in these cells. By contrast, non-hematopoietic cells such as prostate epithelium signal in response to IL-4 or IL-13 via type 2 receptors.

Pre-clinical work has shown a complex and at times contradictory role for IL-4 in tumor immunity. Addition of IL-4 at high concentrations to breast and colorectal cancer cell lines results in inhibition of growth [7]. Several *in vivo* studies involving both immune competent and immunodeficient murine models have also provided support for the anti-tumor activity of this cytokine [8-14]. Following on from these findings, several small and early stage studies were performed to test the toxicity and therapeutic potential of IL-4 in patients with cancer. However, no evidence of efficacy was observed in the majority of cases [15-24], other than a marginal benefit in non-Hodgkin's lymphoma [25]. Administration by the intra-tumoral route has been tested in patients with recurrent head and neck cancer. In one of the treated patients, rapidly advancing disease was observed after cytokine administration, although it remains uncertain whether this was related to IL-4 therapy [26].

A paradoxical role of IL-4 in cancer progression began to emerge following work that was conducted using IL-4 knock-out mice. In light of the considerations described above, one would expect that the absence of IL-4 would favor tumor development. However, in many model systems the exact opposite was seen [27]. Furthermore, IL-4 was found to have a stimulatory effect upon the *in vitro* proliferation of murine prostate and pancreatic tumor cell lines [2,28].

One mechanism by which IL-4 appears to enhance the survival of tumor cells is through a direct anti-apoptotic effect. Studies in colon, breast, lung, fibrosarcoma, prostate and bladder cancer models indicate that IL-4 is not only produced by many cultured tumor cell lines, but that its interaction with IL4-Rα leads to the up-regulation of anti-apoptotic molecules such as cFLIP, PED, FLAME-1 and Bcl-x(L). This anti-apoptotic effect can be abrogated by neutralizing IL-4 antibodies, and is dependent upon downstream STAT-6 signaling [29-32].

In addition to direct actions upon tumor cells, emerging evidence indicates that IL-4 also exerts pro-tumorigenic indirect effects, mediated via the tumor microenvironment. Interleukin-4 producing Th2 cells enhance the metastatic properties of B16 melanoma cell clones [33] and promote macrophage polarization to a pro-tumorigenic M2 phenotype [34]. Tumor-associated IL-4 also polarizes CD8^+^ T-cells to a “type 2” phenotype, leading to further compromised anti-tumor immunity [35].

Taken together, these studies highlight the complex role played by IL-4 in modulating tumor immunity. Broadly speaking however, it appears that supra-physiological production of IL-4 within the tumor microenvironment promotes tumor rejection. By contrast, when lower levels of IL-4 are produced by infiltrating leukocytes, pro-tumor effects are dominant and disease progression ensues.

To unravel the role played by IL-4 in human cancer, serum IL-4 levels have been measured in patients with diverse malignancies. Once again however, results have been conflicting. In several small studies of patients with diverse solid tumors, higher serum IL-4 levels have been correlated with advancing disease [36-43]. Balancing this however, some studies have failed to reproduce these findings [44].

In prostate cancer, chronic inflammation has been implicated as a pathogenic factor [45]. In light of this, two small studies have investigated serum IL-4 levels in prostate cancer patients and have suggested that elevated IL-4 may be linked to disease evolution to castrate resistance [37,38]. However, this finding requires confirmation, not least in light of recent genetic studies suggest that IL-4 plays a tumor-protective role in prostate cancer [46]. To investigate this further, we have analyzed serum levels of IL-4 and the closely related cytokine IL-13 in the largest series studied to date. Furthermore, we have used immunohistochemistry to study the presence and distribution of IL-4 in prostate tumor specimens, making comparison with normal prostate.

## 2. Results and Discussion

### 2.1. Serum IL-4 Levels Are Raised in Patients with Castrate Resistant Prostate Cancer When Compared to Patients with Radically Treatable Disease

Serum samples were collected from 88 patients with prostate cancer. These were distributed into the following groups: (i) Radically treatable group (sampled prior to radical surgery, n = 29); (ii) Androgen-sensitive disease, defined as patients on anti-androgen therapy, with at least two documented drops in serum levels of prostate specific antigen (PSA, n = 29); (iii) Castrate resistant prostate cancer (CRPC) group, defined as patients receiving a lutenising hormone releasing hormone (LHRH) agonist and an anti-androgen with two documented rises in serum PSA (n = 30). The control arm of the study consisted of patients with biopsy-confirmed benign prostatic hypertrophy (BPH group, n = 22). The decision was made to use biopsy-confirmed BPH as the control group since the incidence of sub-clinical prostate cancer is high in men within the age range of the patients recruited. Using this approach, we excluded undetected prostate cancers from the control group.

The age range, serum PSA at time of phlebotomy, and Gleason grade at diagnosis are consistent with what would be expected for the patient group. Ethnicity reflects the population in south-east London from which the patients were recruited. There was a high percentage of black patients in the androgen sensitive group when compared with the other groups (Table 1).

**Figure 1.**
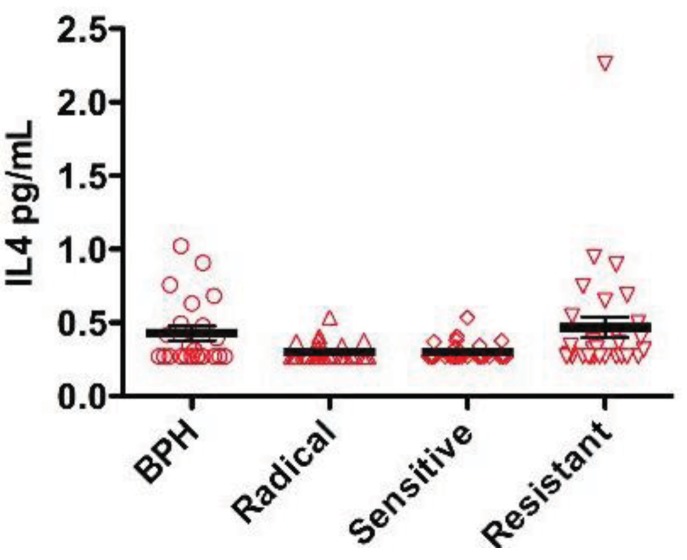

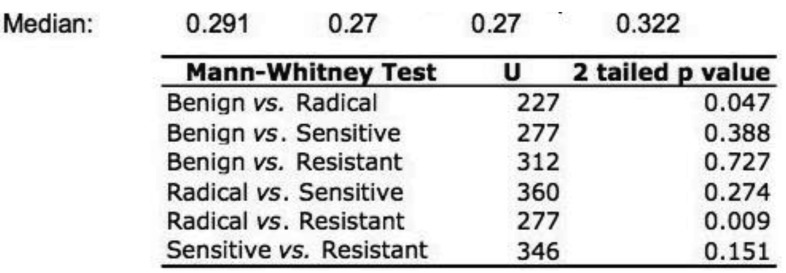
Serum IL-4 levels in patients with benign and malignant prostate disease. Each symbol in the dot plot represents the serum level as measured by ELISA for an individual patient. Results are expressed as median (horizontal line), flanked by the inter-quartile range. Differences were calculated using the Mann-Whitney U Test and are shown in the table as 2-tailed p values.

Both the radically treatable and androgen sensitive groups had a median IL-4 level that was at the lower level of sensitivity of the assay (Figure 1). By contrast, the CRPC group had significantly higher median levels of IL-4 in the serum when compared with the radically treatable group (0.322 *vs.* 0.27 pg/mL, p = 0.009). There was also a modest, statistically significant higher level of IL-4 in BPH patients when compared to the radically treatable group (0.291 *vs.* 0.27 pg/mL, p = 0.047) (Figure 1).

### 2.2. Interleukin-13 Levels are not Significantly Different Between All Four Patient Groups

**Figure 2.**
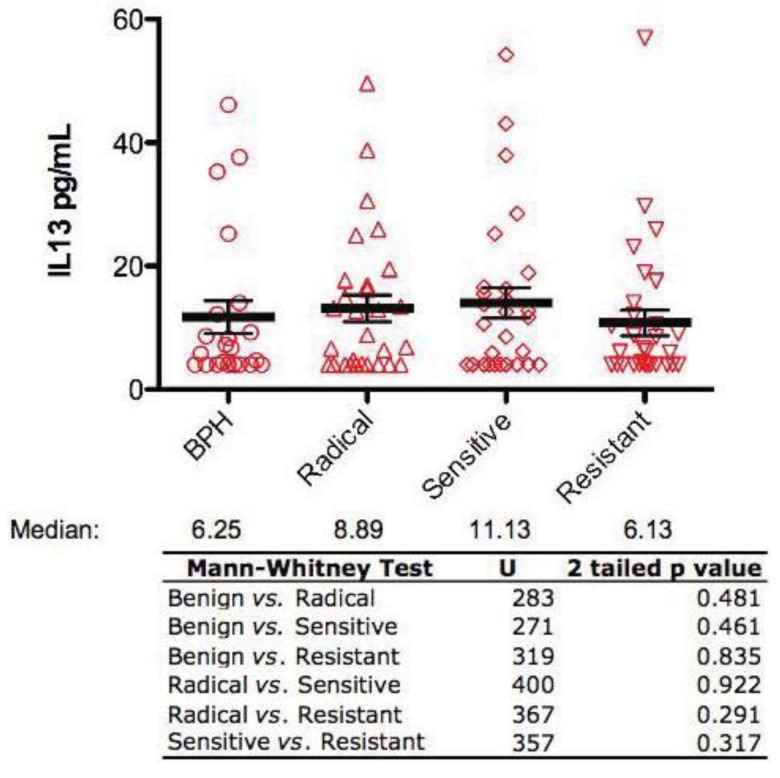
Serum IL-13 levels in patients with benign and malignant prostate disease. Each symbol in the dot plot represents the serum level as measured by ELISA for an individual patient. Results are expressed as median (horizontal line), flanked by the inter-quartile range. Differences were calculated using the Mann-Whitney U Test and are shown in the table as 2-tailed p values.

All serum samples were also analyzed for IL-13 levels by ELISA. This investigation revealed that there were no significant differences in serum IL-13 levels between any of the groups (Figure 2).

### 2.3. Immunohistochemical Detection of Interleukin-4 in Benign and Malignant Prostate Tissue

**Figure 3.**
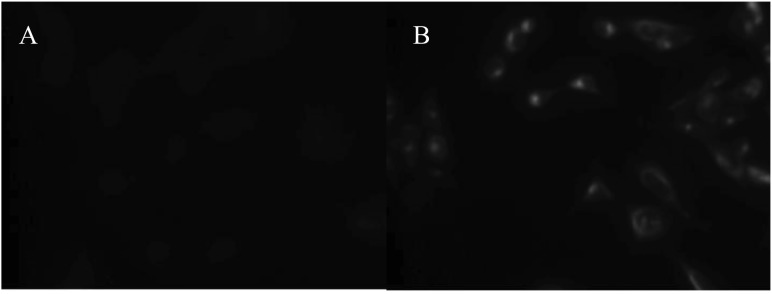
Immunofluorescence staining of paired untransfected (**A**) and human IL-4 transfected (**B**) PG13 cells showing IL-4 specific staining of the transfected cell line.

**Figure 4.**
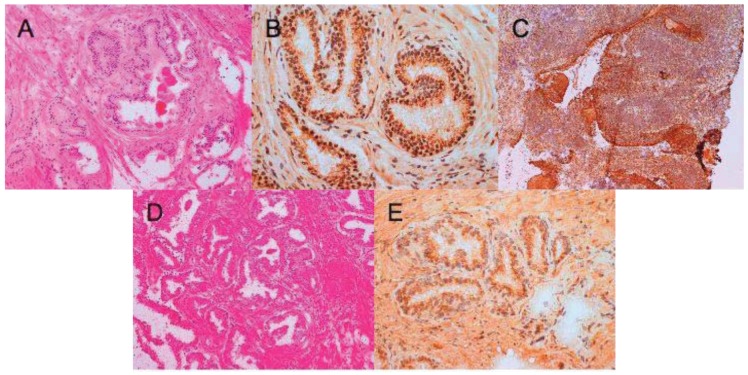
Interleukin-4 immunoreactivity in benign prostate tissue sections. (**A**) Hematoxylin and eosin (H & E) and (**B**) anti-IL-4 staining of benign prostate tissue; (**C**) is a positive control showing anti-IL-4 staining of tonsillar tissue; (**D**) H & E and (**E**) anti-IL-4 staining of prostate *in situ* neoplasia. Shown are two out of a total of six completely benign sections stained.

**Figure 5.**
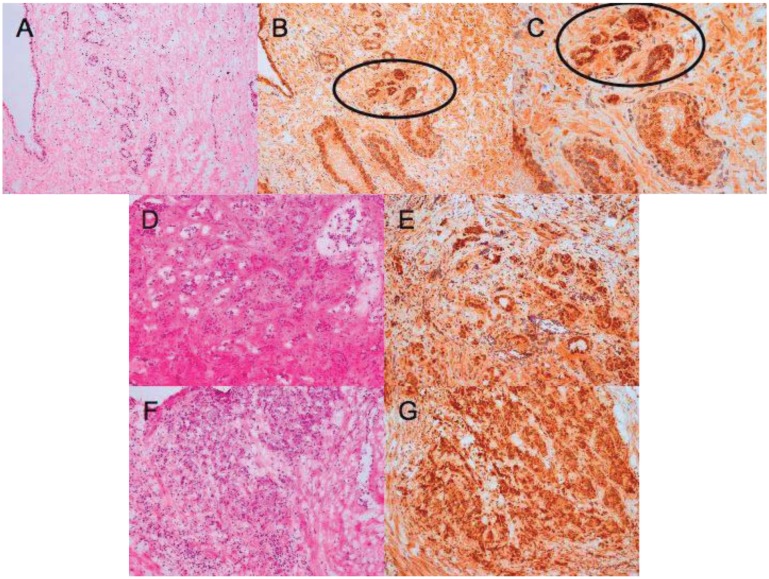
Interleukin-4 immunoreactivity in prostate cancer sections. (**A**) H & E, (**B**) anti-IL-4 and (**C**) anti-IL-4 at higher magnification of a Gleason 3 + 3 prostate cancer showing increased intensity and disorder of anti-IL-4 staining in the malignant glands (circled) when compared with the surrounding normal prostate tissue; (**D**) H & E and (**E**) anti-IL-4 in a Gleason 3 + 4 cancer; (**F**) H & E and (**G**) anti-IL-4 staining in a Gleason 4 + 4 cancer. These images are representative of nine malignant tumors sectioned.

A polyclonal anti-IL-4 antiserum was validated for immunostaining using a human IL-4 transfected cell line (Figure 3) and the reagent was further assessed for use in immunohistochemistry using tonsillar tissue sections (Figure 4C). We used this validated staining technique to examine the distribution of IL-4 in prostate tissue harvested from patients with BPH (Figure 4B), prostate *in situ* neoplasia (PIN) (Figure 4E) and prostate carcinoma (Figure 5). In total fourteen different prostatectomy specimens were sectioned and stained. Six were benign (with two showing areas of PIN). Three were Gleason grade six, two grade seven, two grade eight and one grade ten.

In all cases, IL-4 immunoreactivity was present in glandular structures. In BPH and PIN samples, staining distribution was found to be peri-nuclear and luminal (Figure 4). By contrast, when sections from prostate cancers were subjected to the same anti-IL-4 staining there was loss of luminal polarity. As the Gleason grade of the tumor increased, the intensity of IL-4 staining also increased. In some sections, intense IL-4 immunoreactivity was seen to clearly demarcate areas of malignant glands within normal prostate (see circled malignant glands in Figure 5B,C).

### 2.4. Discussion

Here we report the largest series of prostate cancer patients in whom serum levels of IL-4 and the closely related IL-13 have been measured at all clinically relevant stages of disease. All groups proved to have similar serum levels of IL-13. By contrast, serum IL-4 levels increased proportionately with disease progression. Our data are consistent with those reported in two smaller series, both of which found that IL-4 levels increase as prostate cancer progresses to castrate resistant status [38,39]. This observation is also consistent with evidence that following radical prostatectomy, prognosis is worsened in those patients with a high IL-4 cytokine signature [47]. Mechanistically, these findings may reflect the capacity of IL-4 to activate the androgen receptor in prostate cancer cells, even when ambient androgen levels are very low [1,2].

Our immunohistochemical data is hypothesis generating suggesting a possible role for IL-4 in the pathogenesis of advancing prostate cancer. Compared to benign inflammatory disease, IL-4 staining was less ordered but more intense in tumor tissue and arose from tumor cells themselves. The temporal relationship between serum IL-4 levels and CRPC may be consistent with a role for IL-4 in triggering, or amplifying the castrate switch. The fact that IL-4 but not IL-13 is linked to disease progression raises the possibility that this cytokine acts primarily via the type 1 receptor expressed on infiltrating leukocytes, thereby sculpting the tumor microenvironment. Alternatively (or additionally), tumor-derived IL-4 may act in an autocrine manner upon prostate cancer cells through the type-2 IL-4-receptor [48]. In support of the latter, autocrine IL-4 production has been implicated in the maintenance of colorectal cancer stem cell survival [49]. If the same proves to be the case in prostate cancer, it is tempting to speculate that castrate resistant disease emerges through the enabling action of IL-4 upon prostate cancer stem cells. More extensive and semi-quantitative studies of IL-4 expression in human prostate cancer specimens is warranted.

One unexpected finding of our study was the elevation in serum IL-4 levels in BPH compared to patients with radically treatable prostate cancer. These data are consistent with evidence that BPH is an inflammatory disease [45]. Furthermore, they also agree with the earlier demonstration that IL-4 is upregulated in BPH tissue compared with normal and cancerous prostate cell lines [50]. Taken together, our findings highlight the complex role played by IL-4 in this disease. Indeed, recent genetic evidence supports a protective role for IL-4 over-producer phenotypes against prostate cancer [46]. Consequently, it may be that IL-4 once again serves a dichotomous role in prostate carcinogenesis, exerting opposite effects at early and late stages of disease pathogenesis.

## 3. Experimental Section

### 3.1. Patient Samples

Patients were recruited from the urology and oncology clinics at Guy's and St. Thomas' NHS Foundation Trust, London, UK. All work was approved by the Guy's Hospital Research Ethics Committee, REC number 09/H0804/70 (cytokine study) and 08/H0804/114 (frozen sections).

Whole blood (5 mL) was collected after informed consent into serum separation tubes (BD Vacutainer). After standing at room temperature for 30 min the samples were centrifuged and serum removed and snap frozen in liquid nitrogen. Samples were stored at minus 80 °C until analyzed. Freeze-thaw cycles were minimized.

Tissue blocks stored in liquid nitrogen had been taken from fourteen patients undergoing radical prostatectomy procedures performed for malignant and benign prostate disease at King's College Hospital, London prior to the Human Tissue Act 2004.

### 3.2. Statistical Analysis

Data were not normally distributed. Consequently, statistical analysis was performed using the Mann-Whitney U Test for non-parametric data.

### 3.3. Cytokine Measurement by Enzyme Linked Immunosorbent Assay (ELISA)

For IL-4 quantification in serum, an ultrasensitive IL-4 ELISA Kit (Invitrogen, Paisley, UK). The lower limit of sensitivity is <0.27 pg/mL. There was no cross reactivity with 13 human, six rat and five murine cytokines. For IL-13 quantification in serum, an IL-13 Ready-Set-Go ELISA kit (eBioscience, Hatfield, UK) was used. The lower limit of sensitivity was 4 pg/mL. The IL-4 kit included capture antibody pre-coated 96 well plates. The IL-13 ELISA was carried out using high-binding flat-bottom 96-well ELISA plates, which were coated manually with IL-13 capture antibody (Iwaki, Tokyo, Japan). Assays were performed according to kit manufacturer instructions. Each patient sample was assayed in triplicate with the individual result expressed as a mean.

### 3.4. Interleukin-4 Immunohistochemistry

Frozen blocks from radical prostatectomies performed for prostate cancer were sectioned and stained using haematoxylin and eosin. Immunohistochemical (IHC) staining of validated 5 µm sections was performed with rabbit anti-human IL-4 IgG (ab9622, Abcam, Cambridge, UK), using an Envision™ System with DAB plus (Dako, Carpinteria, CA, USA) as the substrate. Staining protocols were optimized using IL-4 transfected cells and tonsil.

The retroviral packaging cell line PG13 was stably transfected with human interleukin-4 using the retroviral vector SFG. Immunofluorescence was performed using a rabbit anti-human IL-4 IgG (ab9622, Abcam, Cambridge, UK), with secondary polyclonal goat anti-rabbit-FITC antibody staining Goat anti-rabbit IgG FITC-conjugated (Inverness Medical).

## 4. Conclusions

Serum levels of IL-4 are raised in patients with castrate resistant prostate cancer, when compared with patients with organ-confined radically treatable disease. The same is not true of the closely related cytokine IL-13. Using immunohistochemistry, we demonstrated that epithelial cells are the primary source of prostatic IL-4 in both benign and malignant disease. By contrast serum IL-4 levels are elevated in patients with benign inflammatory disease compared to prostate cancer patients amenable to radical therapy. Together, these data are consistent with distinct roles for IL-4 in early and late stages of prostate cancer.

## Figures and Tables

**Table 1.** Clinical and demographic characteristics of the study population.

	**Age (years)**	**PSA (ng/mL)**	**Gleason Grade Mean (STD)**	**Ethnicity**
**BPH n = 22**	Mean 64.4 Range 54–82	Mean 7.1 Range 1.4–26.0	N/A	White: 16
				Black: 4
				Asian: 2
**Radically Treatable n = 29**	Mean 63.8 Range 46–79	Mean 8.9 Range 3.9–34.0	Mean 6.9 (1.20)	White: 22
				Black: 6
				Asian: 1
**Androgen Sensitive n = 29**	Mean 74.8 Range 54–85	Mean 3.5 Range 0.03–18.4	Mean 7.5 (2.34)	White: 17
				Black: 12
				Asian: 0
**Castrate Resistant n = 30**	Mean 72.5 Range 49–84	Mean 234.3 Range 0.1–1197	Mean 7.8 (1.01)	White: 25
				Black: 4
				Asian: 1

N/A: not applicable

## References

[1] Lee S.O., Lou W., Nadiminty N., Lin X., Gao A.C. (2005). Requirement for NF-(kappa)B in interleukin-4-induced androgen receptor activation in prostate cancer cells. Prostate.

[2] Lee S.O., Pinder E., Chun J.Y., Lou W., Sun M., Gao A.C. (2008). Interleukin-4 stimulates androgen-independent growth in LNCaP human prostate cancer cells. Prostate.

[3] Galizzi J.P., Zuber C.E., Harada N., Gorman D.M., Djossou O., Kastelein R., Banchereau J., Howard M., Mijajima A. (1990). Molecular cloning of a cDNA encoding the human interleukin 4 receptor. Int. Immunol..

[4] Russell S.M., Keegan A.D., Harada N., Nakamura Y., Noguchi M., Leland P., Friedmann M.C., Miyajima A., Puri R.K., Paul W.E. (1993). Interleukin-2 receptor gamma chain: A functional component of the interleukin-4 receptor. Science.

[5] Aman M.J., Tayebi N., Obiri N.I., Puri R.K., Modi W.S., Leonard W.J. (1996). cDNA cloning and characterization of the human interleukin 13 receptor alpha chain. J. Biol. Chem..

[6] Palmer-Crocker R.L., Hughes C.C., Pober J.S. (1996). IL-4 and IL-13 activate the JAK2 tyrosine kinase and Stat6 in cultured human vascular endothelial cells through a common pathway that does not involve the gamma c chain. J. Clin. Invest..

[7] Toi M., Bicknell R., Harris A.L. (1992). Inhibition of colon and breast carcinoma cell growth by interleukin-4. Cancer Res..

[8] Golumbek P.T., Lazenby A.J., Levitsky H.I., Jaffee L.M., Karasuyama H., Baker M., Pardoll D. (1991). Treatment of established renal cancer by tumor cells engineered to secrete interleukin-4. Science.

[9] Noffz G., Qin Z., Kopf M., Blankenstein T. (1998). Neutrophils but not eosinophils are involved in growth suppression of IL-4-secreting tumors. J. Immunol..

[10] Pericle F., Giovarelli M., Colombo M.P., Ferrari G., Musiani P., Modesti A., Cavallo F., di Pierro F., Novello F., Forni G. (1994). An efficient Th2-type memory follows CD8^+^ lymphocyte-driven and eosinophil-mediated rejection of a spontaneous mouse mammary adenocarcinoma engineered to release IL-4. J. Immunol..

[11] Pippin B.A., Rosenstein M., Jacob W.F., Chiang Y., Lotze M.T. (1994). Local IL-4 delivery enhances immune reactivity to murine tumors: Gene therapy in combination with IL-2. Cancer Gene Ther..

[12] Rodolfo M., Zilocchi C., Accornero P., Cappetti B., Arioli I., Colombo M.P. (1999). IL-4-transduced tumor cell vaccine induces immunoregulatory type 2 CD8 T lymphocytes that cure lung metastases upon adoptive transfer. J. Immunol..

[13] Tepper R.I., Pattengale P.K., Leder P. (1989). Murine interleukin-4 displays potent anti-tumor activity *in vivo*. Cell.

[14] Yu J.S., Wei M.X., Chiocca E.A., Martuza R.L., Tepper R.I. (1993). Treatment of glioma by engineered interleukin 4-secreting cells. Cancer Res..

[15] Atkins M.B., Vachino G., Tilg H.J., Karp D.D., Robert N.J., Kappler K., Mier J. (1992). Phase I evaluation of thrice-daily intravenous bolus interleukin-4 in patients with refractory malignancy. J. Clin. Oncol..

[16] Margolin K., Aronson F.R., Sznol M., Atkins M.B., Gucalp R., Fisher R.I., Sunderland M., Doroshow J.H., Ernest M.L., Mier J.W. (1994). Phase II studies of recombinant human interleukin-4 in advanced renal cancer and malignant melanoma. J. Immunother. Emphasis Tumor Immunol..

[17] Miles S.A., Testa M., Huang J., Wade M., Carden J., Scadden D.T. (2002). Lack of antitumor activity and intolerance of interleukin-4 in patients with advanced HIV disease and Kaposi's sarcoma. J. Interferon Cytokine Res..

[18] Sosman J.A., Fisher S.G., Kefer C., Fisher R.I., Ellis T.M. (1994). A phase I trial of continuous infusion interleukin-4 (IL-4) alone and following interleukin-2 (IL-2) in cancer patients. Ann. Oncol..

[19] Stadler W.M., Rybak M.E., Vogelzang N.J. (1995). A phase II study of subcutaneous recombinant human interleukin-4 in metastatic renal cell carcinoma. Cancer.

[20] Taylor C.W., LeBlanc M., Fisher R.I., Moore D.F., Roach R.W., Elias L., Miller T.P. (2000). Phase II evaluation of interleukin-4 in patients with non-Hodgkin's lymphoma: A Southwest Oncology Group trial. Anticancer Drugs.

[21] Tulpule A., Joshi B., DeGuzman N., Espina B.M., Mocharnuk R., Prakash O., Templeton D., Levine A.M., Gill P.S. (1997). Interleukin-4 in the treatment of AIDS-related Kaposi's sarcoma. Ann. Oncol..

[22] Whitehead R.P., Lew D., Flanigan R.C., Weiss G.R., Roy V., Glode M.L., Dakhil S.R., Crawford E.D. (2002). Phase II trial of recombinant human interleukin-4 in patients with advanced renal cell carcinoma: A Southwest Oncology Group study. J. Immunother..

[23] Whitehead R.P., Unger J.M., Goodwin J.W., Walker M.J., Thompson J.A., Flaherty L.E., ondak V.K. (1998). Phase II trial of recombinant human interleukin-4 in patients with disseminated malignant melanoma: A Southwest Oncology Group study. J. Immunother..

[24] Wiernik P.H., Dutcher J.P., Yao X., Venkatraj U., Falkson C.I., Rowe J.M., Cassileth P.A. (2010). Phase II study of interleukin-4 in indolent B-cell non-Hodgkin lymphoma and B-cell chronic lymphocytic leukemia: A study of the Eastern Cooperative Oncology Group (E5Y92). J. Immunother..

[25] Kurtz D.M., Tschetter L.K., Allred J.B., Geyer S.M., Kurtin P.J., Putnam W.D., Rowland K.M., Wiesenfeld M., Soori G.S., Tenglin R.C. (2007). Subcutaneous interleukin-4 (IL-4) for relapsed and resistant non-Hodgkin lymphoma: A phase II trial in the North Central Cancer Treatment Group, NCCTG 91-78-51. Leuk. Lymphoma.

[26] Werkmeister R., Fillies T., Gaertner C., Joos U., Berdel W.E. (2005). A clinical phase I/II trial of rhIL-4 applied topically in patients with oral squamous cell carcinomas to assess safety and therapeutic activity. Oncol. Rep..

[27] Stremmel C., Greenfield E.A., Howard E., Freeman G.J., Kuchroo V.K. (1999). B7-2 expressed on EL4 lymphoma suppresses antitumor immunity by an interleukin 4-dependent mechanism. J. Exp. Med..

[28] Prokopchuk O., Liu Y., Henne-Bruns D., Kornmann M. (2005). Interleukin-4 enhances proliferation of human pancreatic cancer cells: Evidence for autocrine and paracrine actions. Br. J. Cancer.

[29] Conticello C., Pedini F., Zeuner A., Patti M., Zerilli M., Stassi G., Messina A., Peschle C., de Maria R. (2004). IL-4 protects tumor cells from anti-CD95 and chemotherapeutic agents via up-regulation of antiapoptotic proteins. J. Immunol..

[30] Li Z., Jiang J., Wang Z., Zhang J., Xiao M., Wang C., Lu Y., Qin Z. (2008). Endogenous interleukin-4 promotes tumor development by increasing tumor cell resistance to apoptosis. Cancer Res..

[31] Todaro M., Lombardo Y., Francipane M.G., Alea M.P., Cammareri P., Iovino F., di Stefano A.B., di Bernardo C., Agrusa A., Condorelli G. (2008). Apoptosis resistance in epithelial tumors is mediated by tumor-cell-derived interleukin-4. Cell Death Differ..

[32] Li B.H., Yang X.Z., Li P.D., Yuan Q., Liu X.H., Yuan J., Zhang W.J. (2008). IL-4/Stat6 activities correlate with apoptosis and metastasis in colon cancer cells. Biochem. Biophys. Res. Commun..

[33] Kobayashi M., Kobayashi H., Pollard R.B., Suzuki F. (1998). A pathogenic role of Th2 cells and their cytokine products on the pulmonary metastasis of murine B16 melanoma. J. Immunol..

[34] DeNardo D.G., Barreto J.B., Andreu P., Vasquez L., Tawfik D., Kolhatkar N., Coussens L.N. (2009). CD4(+) T cells regulate pulmonary metastasis of mammary carcinomas by enhancing protumor properties of macrophages. Cancer Cell.

[35] Apte S.H., Groves P., Olver S., Baz A., Doolan D.L., Kelso A., Kienzle N. (2010). IFN-gamma inhibits IL-4-induced type 2 cytokine expression by CD8 T cell *in vivo* and modulates the anti-tumor response. J. Immunol..

[36] Filella X., Alcover J., Zarco M.A., Beardo P., Molina R., Ballesta A.M. (2000). Analysis of type T1 and T2 cytokines in patients with prostate cancer. Prostate.

[37] Perambakam S.M., Srivastava R., Peace D.J. (2005). Distinct cytokine patterns exist in peripheral blood mononuclear cell cultures of patients with prostate cancer. Clin. Immunol..

[38] Takeshi U., Sadar M.D., Suzuki H., Akakura K., Sakamoto S., Shimbo M., Suyama T., Imamoto T., Komiya A., Yukio N. (2005). Interleukin-4 in patients with prostate cancer. Anticancer Res..

[39] Wise G.J., Marella V.K., Talluri G., Shirazian D. (2000). Cytokine variations in patients with hormone treated prostate cancer. J. Urol..

[40] Onishi T., Ohishi Y., Goto H., Tomita M., Abe K. (2001). An assessment of the immunological status of patients with renal cell carcinoma based on the relative abundance of T-helper 1- and -2 cytokine-producing CD4^+^ cells in peripheral blood. BJU Int..

[41] Onishi T., Ohishi Y., Imagawa K., Ohmoto Y., Murata K. (1999). An assessment of the immunological environment based on intratumoral cytokine production in renal cell carcinoma. BJU Int..

[42] Pellegrini P., Berghella A.M., Del Beato T., Cicia S., Adorno D., Casciani C.U. (1996). Disregulation in TH1 and TH2 subsets of CD4^+^ T cells in peripheral blood of colorectal cancer patients and involvement in cancer establishment and progression. Cancer Immunol. Immunother..

[43] Yamazaki K., Yano T., Kameyama T., Suemitsu R., Yoshino I., Sugio K. (2002). Clinical significance of serum TH1/TH2 cytokines in patients with pulmonary adenocarcinoma. Surgery.

[44] Lissoni P., Merlini D., Pirato D., Meregalli S. (1997). Interleukin-4 blood concentrations in early and metastatic human solid neoplasms. Int. J. Biol. Markers.

[45] Kramer G., Mitteregger D., Marberger M. (2007). Is benign prostatic hyperplasia (BPH) an immune inflammatory disease. Eur. Urol..

[46] Tindall E.A., Severi G., Hoang H.N., Ma C.S., Fernandez P., Southey M.C., English D.R., Hopper J.L., Heyns C.F., Tangye S.G. (2010). Comprehensive analysis of the cytokine-rich chromosome 5q31.1 region suggests a role for IL-4 gene variants in prostate cancer risk. Carcinogenesis.

[47] Quatan N., Meyer B., Bailey M., Pandha H. (2006). Persistently high levels of immunosuppressive cytokines in patients after radical prostatectomy. Prostate Cancer Prostatic Dis..

[48] Ammirante M., Luo J.L., Grivennikov S., Nedospasov S., Karin M. (2010). B-cell-derived lymphotoxin promotes castration-resistant prostate cancer. Nature.

[49] Todaro M., Alea M.P., di Stefano A.B., Cammareri P., Vermeulen L., Iovino F., Tripodo C., Russo A., Gulotta G., Medema J.P. (2007). Colon cancer stem cells dictate tumor growth and resist cell death by production of interleukin-4. Cell Stem Cell.

[50] Kramer G., Steiner G.E., Handisurya A., Stix U., Haitel A., Knerer B., Gessl A., Lee C., Marberger M. (2002). Increased expression of lymphocyte-derived cytokines in benign hyperplastic prostate tissue, identification of the producing cell types, and effect of differentially expressed cytokines on stromal cell proliferation. Prostate.

